# Psychometric properties of the Persian version of postpartum distress measure scale

**DOI:** 10.1186/s12888-020-02497-0

**Published:** 2020-02-27

**Authors:** Zahra Bakht Shokuhi, Fatemeh Ranjbar, Sevil Hakimi, Rogayeh Bahri, Saeideh Ghaffarifar

**Affiliations:** 1 Research center of psychiatry and behavioral sciences, Tabriz University of Medical Sciences, Tabriz, Iran; 2 School of Nursing and Midwifery, Tabriz University of Medical Sciences, Tabriz, Iran; 3 Department of clinical psychology, Fabric branch, Islamic Azad University, Tabriz, Iran; 4 Medical Education Research Centre, Health Management and Safety Promotion Research Institute, Tabriz University of Medical Sciences, Tabriz, Iran

**Keywords:** Postpartum measure scale, Psychometric properties, Persian

## Abstract

**Background:**

The purpose of the present study was to determine psychometric properties of the Persian version of Postpartum Distress Measure Scale (PDM Scale).

**Methods:**

In this psychometric explorative study, the data were collected using a questionnaire containing demographic information, PDM Scale, and Depression and Anxiety Stress Scale-21 (DASS-21). The content, face and construct validity of the questionnaire was examined with participation of ten experts, 10 and 150 women referring to health care centers, who were under common care during their postpartum period, respectively. The concurrent validity of the tool was evaluated using DASS-21. The reliability of the items was evaluated with the participation of 30 women, calculating Cronbach’s alpha coefficient and intra-class correlation coefficient.

**Results:**

The Content Validity Index, Content Validity Ratio and Impact Score of the Persian version of the PDM were 0.94, 0.73, and 2.97, respectively. The ten items of the questionnaire were loaded in two factors (general distress and obsessive compulsive symptoms subscale). Those two factors explained 50.78% of the total variance of women’s distress. Internal consistency of the items and stability of the results were confirmed by Cronbach’s alpha of 0.72 and Intra-class correlation coefficient of 0.75.

**Conclusion:**

According to the study results, the Persian version of PDM Scale has acceptable psychometric properties. Care providers and researchers can use it as a tool for screening anxiety, depression and obsessive-compulsive disorder in women.

## Background

Postpartum is a very important and stressful period in women and their families” life. During this period, some degrees of worry, anxiety and low mood are normal among women, especially among nulliparous women or the women with unplanned pregnancy. Emotional problems in postpartum period are not limited to depression and can also include symptoms of anxiety and obsessive-compulsive disorders [1].

There are usually some degrees of worry about childbirth, adaptation with postpartum conditions and the birth of a normal baby; however, a percentage of women are more worried and experience extreme abnormal fear [2]. In most cases, the main causes of such an abnormal fear are related to the nutritional problems of the baby [3, 4].

On the other hand, physicians who investigate only depression in mothers may be unaware of the distress that women experience [5].

Psychiatric disorders bring undesired consequences for mother and baby, including premature births and low birth weight at birth [5, 6]. Moreover, children of depressed mothers, who have not been treated during or after pregnancy, are at risk of attachment disorders, poor relationship with the mother and developmental problems [7–9]. For mothers with severe psychiatric disorder, more suicide rates are reported, too [10].

In a study by Glasheen and colleagues (2015), the prevalence of severe psychological distress (SPD) in pregnant women was 6.4% in the past month and in the first trimester and 3.9 in the third trimester. In postpartum women, the rate was 4.6% in the first two months. It was 6.9% for 3–5 months later [10]. In a study by Ahluwalia and associates (2004), the prevalence of SPD was 6.7%, and the prevalence of recent psychological distress; depression; worry and anxiety; and inadequate rest was reported as 12.3, 9.9, 18.4, and 34.3%, respectively [11]. In a study by Skari and colleagues (2002), 127 pregnant women and 122 fathers showed that the prevalence of psychological distress in pregnant women and fathers was 37 and 13%, respectively.

Single parenthood, multi-parity and history of difficult delivery are the risk factors, which contribute to psychological distress [12].

Currently, there are several scales for evaluating patients’ anxiety levels. The most important of which are Beck Anxiety Inventory, *Spielberger* Trait Anxiety Inventory and *Edinburgh* Postnatal *Depression Scale* (EPDS), or the 7-item Generalized Anxiety Inventory (GAD-7) [13]. However, clinical manifestations of depression, generalized anxiety disorder, and obsessive-compulsive disorder are not simultaneously screened by these questionnaires. In practice, such a problem leads to less desired diagnosis of postpartum mental distress, lower rate of referrals and ineffective therapeutic approaches. In this regard, a study by Goodman and collegues (2010) found that 17% of the patients had symptoms of depression or anxiety disorders 6 weeks after delivery; however, only one quarter of the patients were screened; two percentage of them were referred and received adequate treatment [14].

Allison and associates (2011) developed and validated postpartum distress measure scale. This scale measures the symptoms of postpartum distress. This is the first postpartum screening tool, which is specific for simultaneous screening of anxiety and depression symptoms. With this scale, symptoms of generalized distress and obsessive-compulsive disorder are assessed reliably. The use of this Post-partum Distress Measure (PDM) Scale increases the possibility of diagnosing any significant clinical symptoms, including clinical history of depression, generalized anxiety disorder, and obsessive-compulsive disorder, which is reported in two-thirds of postpartum women [15].

Up to the time of this research and to the best of our knowledge and literature review, there was no Persian scale for evaluating postpartum distress. Therefore, the present study was aimed to translate and adapt the original version of PDM Scale and determine the psychometric properties of its Persian version.

## Methods

This psychometric explorative study was conducted in health centers, affiliated to Tabriz University of Medical Sciences, from April 2015 to March 2016. The data collection tool was the English version of the Postpartum Distress Measure Scale (PDM Scale). The ethics approval was obtained from university review board (No. IR.TBZMED.REC 1395.660). Permission for translation and adaptation of the original questionnaire into Persian language was obtained from the authors of the English version of PDM Scale through an e-mail.

Translation and adaptation were done using Forward-Backward translation approach. For this purpose, one psychiatrist and one reproductive health specialist separately translated the original version of the questionnaire into Persian. The two translated versions were compared and the differences and ambiguities were identified and resolved. Then, the final Persian version was translated into English by one bilingual translator who did not participate already in the study. Finally, in a committee by the presence of the translators and the research team, the English version of the questionnaire was compared with the original version and all semantic problems were resolved.

### Statistical analysis

#### Assessment of the content validity

After the stages of translation and preparation of the Persian version of the questionnaire, the content and face validity of the questionnaire were evaluated in both qualitative and quantitative ways. For so doing, ten experts, including eight psychiatrists, one reproductive health specialist and one psychologist, provided written comments on how to observe the grammar, the use of proper words, the placement of the items in their proper place and appropriate scoring of the items. In order to assess content validity, Content Validity Ratio (CVR) and Content Validity Index (CVI) were calculated.

In order to determine**”**CVR, the experts were asked to examine each item based on 3-part Likert scale (it is necessary; useful, but not necessary; not necessary).

To calculate CVI, relevance, simplicity and clarity of the items were examined, using a four-part Likert scale (for instance: 1 = not relevant, 2 = relatively relevant, 3 = relevant and 4 = completely relevant).

Using the formula of $VR=\frac{\mathrm{N}\mathrm{e}-\mathrm{N}/2}{\mathrm{N}/2}$, CVR was calculated. Lawseh table was used to decide the values. Since 10 specialists completed the questionnaires, any score more than 0.62 was considered to be acceptable for confirming the content validity of each item.

CVI was calculated by the formula of C $VI=\frac{\mathrm{The}\ \mathrm{number}\ \mathrm{of}\ \mathrm{specialists}\ \mathrm{who}\ \mathrm{gave}\ 3\ \mathrm{and}\ 4\ \mathrm{for}\ \mathrm{the}\ \mathrm{items}}{\mathrm{N}}$ and CVIs over 0.79 were considered acceptable.

#### Assessment of the face validity

Face validity of the questionnaire was evaluated by a panel of experts, including eight psychiatrists, one reproductive health specialist and one psychologist and ten women with postpartum distress, in two qualitative and quantitative methods. For qualitative evaluation, face-to-face interviews were conducted with ten participating women. Difficulty in understanding the words and the possibility of any misunderstandings and ambiguity in the meaning of the phrases were investigated. Those ten participating women were randomly selected from 5 health centers (two women from each center). For quantitative evaluation, the experts evaluated the degree of appropriateness of each item, based on a 5-point Likert scale (very important = 5, somehow important = 4, moderately important = 3, slightly important = 2, and not important = 1). In doing so, the impact score of each item was calculated. Impact scores (IS) were calculated by the following formula: Impact Score = Frequency (%) × Importance. Impact score of each item had to be above 1.5 and the items with IS over 1.5 were considered acceptable in terms of face validity.

#### Assessment of the construct validity

Construct validity of the questionnaire was studied by exploratory factor analysis. Criterion-related validity (concurrent validity) was evaluated through correlation test between the results of two questionnaires of DASS-21 and PDM Scale. Factor analysis was performed to reduce the data, explain and clarify the theoretical structure of the questionnaire. Sampling adequacy was studied by KMO (Kaiser-Meyer-Olkin) Measure and Bartlett’s test of Sphericity. The data were extracted by principal component method and the rotation of the factors by Varimax method.

In order to assess the construct validity of the questionnaire, random and multi-stage sampling was done. After the random selection of 15 centers from the list of all health centers in Tabriz, the cases of women who were in postpartum period were examined and from each center, a number of cases were randomly selected based on the total number of available cases.

In this phase, 150 women with postpartum distress were recruited. As the women with postpartum distress have similar clinical manifestations all around the country, the women in Tabriz were considered to be representative of other women in Iran. In Iran, governmental facilities are provided for all women, who have just given birth to their children and they are regularly visited at health centers after delivery. As those facilities are provided for free, almost all women refer to health centers. So, access to the women with postpartum distress is highly provided at those health centers. In other words, employing participants from health centers increased the feasibility of our study.

Women who announced their written informed consent participated in the study. The questionnaire was completed for all referring women who had symptoms or signs of distress. Women with a probable diagnosis were visited by a psychiatrist and those who were approved to suffer from any degrees of postpartum distress were recruited in the present study.

The inclusion criteria were: aged 19–40 years and being referred during the first year after delivery. Exclusion criteria included: having a diagnosed psychiatric disease or history of high risk pregnancy, including gestational diabetes, pre-eclampsia, fetal growth retardation, twin pregnancy, instrumental delivery and fetal abnormalities.

Data collection tools in this study included three questionnaires. All three questionnaires were completed for all participants. In general, for those who did not have reading and writing literacy, questions were read by the main researcher and the responses given by the participants were re-checked and marked by her.

The first questionnaire included baseline characteristics of the participants: including their age, marital status, job, education level and their family economic status.

The second questionnaire was the Persian version of PDM scale. Both the face and content validity of this questionnaire is evaluated in the present study. This questionnaire has 10 items. The completion time of each questionnaire was five minutes. The participating women, by assigning a score in the range of 0 to 3, evaluated the severity of each of the symptoms they felt. A four- point Likert Scale (not at all = 0, low = 1, medium = 2, and high = 3) was used for scoring each item. Except for the items number one, six and ten, other items were reversely scored. The minimum and maximum scores are zero and 48, respectively. The higher the score, the greater the distress [15].

#### Concurrent validity assessment

DASS-21 was the third questionnaire, which was used in this study. It was used to determine the concurrent validity of the Persian version of Postpartum Distress Measure. This scale provides valid and reliable assessment of the three constructs of depression, anxiety and stress [16]. The internal consistency and validity of DASS-21 is within an acceptable to excellent range. According to some studies, its 21-item version has several advantages over its full 42-item version, so its use is preferred to the use of its full version [15]. The main DASS-21 questionnaire consists of three subscales of depression, anxiety and stress, and a total of 21 items. Each sub-scale consists of seven items. Its items address the status of patients’ symptoms in the last week. Each item has a score of 0 to 3(very true to me = 3, it is pretty true to me = 2, to some extent true to me = 1, and by no means true to me = 0). For each sub-scale, the range of scores can be from 0 to 21. In the 21-question version, the sub-scales are multiplied by two. The higher the scores, the higher the severity of depression, anxiety and stress [15]. That questionnaire was translated into Persian by Asghari and colleagues in 2008. Its Persian version has appropriate psychometric properties. The internal consistency coefficients of depression, anxiety and stress scales were 0.93, 0.90, and 0.92, respectively. Intra-class correlation coefficients of depression, anxiety and stress subscales were 0.84, 0.89, and 0.90, respectively [17].

#### Reliability assessment

The internal consistency of the items was studied by calculating Cronbach’s alpha coefficient. Stability of the results was examined applying test-retest method and calculating intra-class correlation coefficient (ICC). For this purpose, the questionnaire was completed twice by 30 women with postpartum distress during a 10 day interval.

#### Quality supervision of the study

In order to implement the quality supervision of the study, the research questionnaire was administered to the meticulous and caring participants, whether the experts or the women with postpartum distress. Content Validity Index (CVI) and Content Validity Ratio (CVR) were determined by two different groups of experts. In addition, the data of the completed questionnaires were manually checked for any careless completing or responding before the data were analyzed.

As all questionnaires were completed in the presence of health care providers, there was no missing data in this study; however, in case of any missing data, it was planned to perform sensitivity analyses, missing data patterns analysis or imputation and of course reporting.

Statistical analysis was performed using SPSS software, version 18 (IBM, Armonk, NY, USA).

## Results

In this study, 150 mothers participated in their postpartum period. The mean age of the participants was 29.92 ± 5.45 years. 85.3% of the participants were households, 75.3% of the women had no history of abortion, and 53.3% of the new-borns were girls. 98% of women were satisfied with their child gender and 87.3% of mothers were satisfied with their pregnancy. Spouse’s support for participants was high and moderate among 77.3 and 17.3% of the women, respectively. Other baseline characteristics of the participating women are presented in Table 1.

**Table 1:** Baseline characteristics of the women participating in the psychometric study of the Persian version of Postpartum Distress Measure scale (*n* = 150)

Variable		
		**Mean ± SD (Min to Max)**
Age		29.92 ± 5.45 (19 to40)
Spouse age		34.51 ± 5.75 (22 to 55)
		**Frequency (percentage)**
Education level	**Illiterate**	22 (14.6)
	**High school**	58 (38.7)
	**University**	70 (46.7)
Spouse job	**Unemployed**	1 (0.7)
	**Employed**	29 (19.3)
	**Worker**	34 (22.7)
	**Self-employed**	52 (34.6)
	**Driver**	24 (16)
	**Others**	10 (6.7)
Spouse education Level	**Illiterate**	1 (0.7)
	**High school**	63 (42)
	**University**	86 (57.3)

The Content Validity Index, Content Validity Ratio and Impact Score of the Persian version of the PDM were 0.94, 0.73, and 2.97, respectively.

**Fig. 1:**
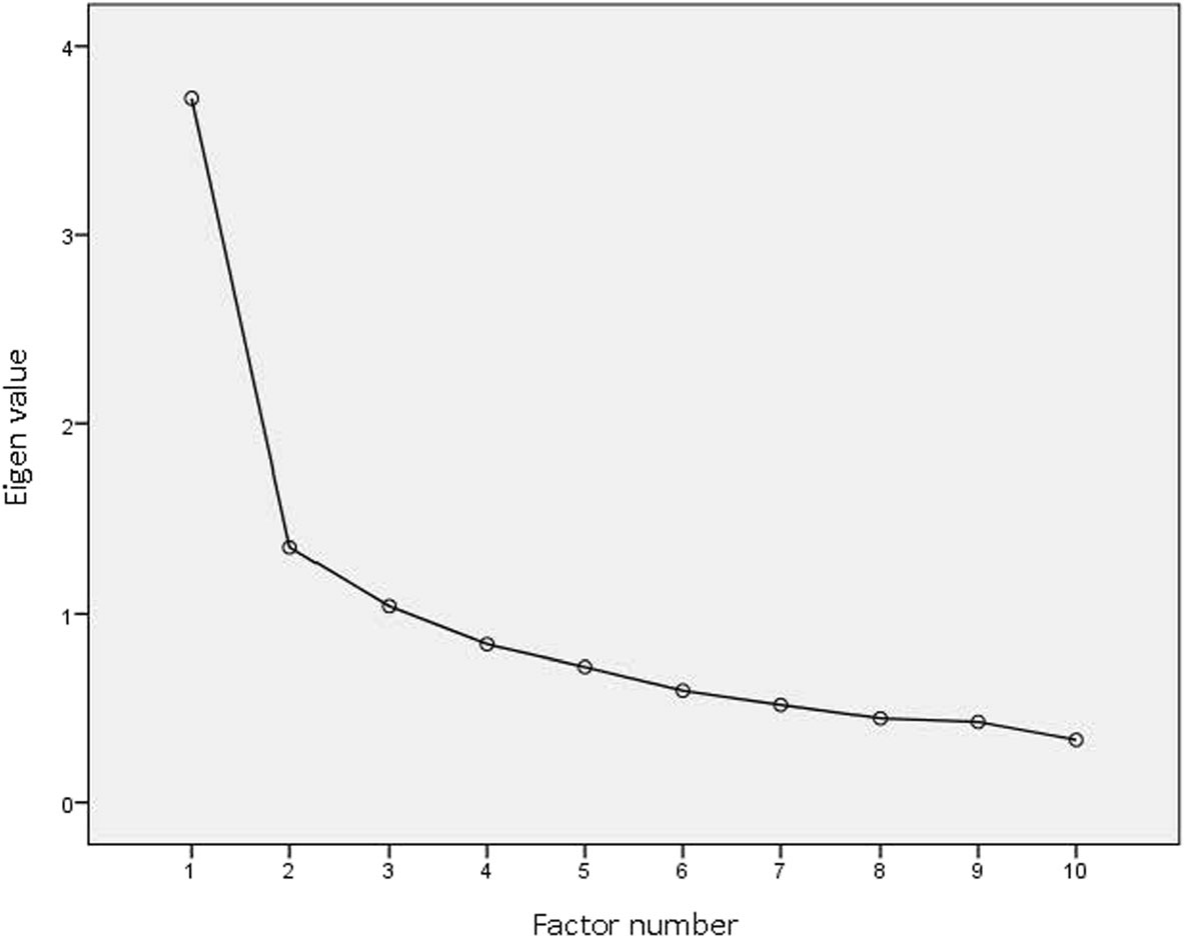
Factor load scree plot of the items for determining the number of extracted factors of the Persian version of Postpartum Distress Measure Scale

The results for the content and face validity of the items are presented in Table 2. The communalities of 10 items of PDM Scale are shown in Table 3. Ten items with eigenvalues greater than one were extracted. These 10 items in two factors were able to explain 50.78% of the total variance. All items of the questionnaire were loaded in two factors. Factor load scree plot is shown on Fig. 1.The naming of each of the factors was agreed based on the original version of the questionnaire.

**Table 2:** The results for the content and face validity of the Persian version of Postpartum Distress Measure Scale

Item	Content Validity Index			Content Validity Ratio	Impact Score
	Relevance	Clarity	Simplicity		
I feel sad and hopeless	0.8	0.9	1	0.8	3.8
I am crying more than usual	0.9	0.9	0.8	0.8	1.5
I cannot make decisions or concentrate	0.9	1	1	0.6	1.7
I feel overwhelmed	0.9	1	1	0.8	4.7
I’m afraid I will never feel better	1	1	0.8	0.7	3.7
I think about taking my own life	0.9	0.9	0.8	0.7	2.5
I have recurring thoughts about harm coming to my baby, my family, or myself	1	1	1	0.7	3.2
I have recurring thoughts about my baby getting sick or having some kind of problem	1	1	1	0.8	2.5
I check on my baby multiple times throughout the night	1	0.9	0.8	0.7	3.4
I have thoughts about my baby that scare me	1	1	1	0.7	2.7
The mean of the measures for the whole questionnaire	0.94	0.96	0.92	0.73	2.97

**Table 3:** Communalities of the 10 items of the Persian version of Postpartum Distress Measure Scale

Item	Communality
I feel sad and hopeless	0.537
I am crying more than usual	0.460
I cannot make decisions or concentrate	0.570
I feel overwhelmed	0.455
I’m afraid I will never feel better	0.617
I think about taking my own life	0.758
I have recurring thoughts about harm coming to my baby, my family, or myself	0.223
I have recurring thoughts about my baby getting sick or having some kind of problem	0.433
I check on my baby multiple times throughout the night	0.476
I have thoughts about my baby that scare me	0.548

The initial and rotated eigenvalues of two-factor PDM Scale are shown in Table 4.

**Table 4:** Initial and rotated eigenvalues of 2-factors of the Persian version of Postpartum Distress Measure Scale

Factor	Initial eigenvalue			Total rotated square		
	Total	Variance %	Cumulative %	Total	Variance %	Cumulative %
1	3.72	37.24	37.24	2.95	29.59	29.59
2	1.35	13.53	50.78	2.11	21.19	50.78

According to the results of exploratory factor analysis, two primary factors were extracted. The first factor called **“**General Distress**”**, explained 37.24% of the variance. The second factor called “obsessive-compulsive” explained 13.53% of the variance. The factor load for the items is shown in Table 5. Considering the importance of suicidal thoughts (item number seven), the research team decided not to eliminate item seven.

**Table 5:** Factor load of items of the Persian version of Postpartum Distress Measure Scale

Item	Factor load		
	1	2	3
I feel sad and hopeless	0.892	0.100	–
I am crying more than usual	0.649	–	–
I cannot make decisions or concentrate	0.727	–	–
I feel overwhelmed	0.440	0.480	–
I’m afraid I will never feel better	0.784	–	–
I think about taking my own life	0.532	0.680	–
I have recurring thoughts about harm coming to my baby, my family, or myself	0.395	0.448	–
I have recurring thoughts about my baby getting sick or having some kind of problem	0.281	0.660	0.425
I check on my baby multiple times throughout the night	0.395	0.590	–
I have thoughts about my baby that scare me	–	0.339	–

At the stage of determining criterion-related (concurrent) validity, the correlation between general distress and anxiety, depression and stress was 0.531, 0.422 and 0.460, respectively (*P* = 0.001). The correlation coefficient between obsessive compulsive and anxiety, depression or stress was 0.412, 0.396 and 0.562 (*P* = 0.001).

Intra-class correlation coefficient for the construct of general distress was 0.68.It was 0.73 for the construct of obsessive compulsive and 0.75 for the whole questionnaire (*P* = 0.001).

In order to investigate the homogeneity of constructs, Cronbach’s alpha coefficient was calculated. Cronbach’s alpha for the factor of general distress was 0.60. It was 0.62 for the factor of obsessive compulsive and 0.72 for the PDM scale in all.

Sampling adequacy was confirmed by KMO of 0.811 and significance of 0.000 of the Bartlett’s test of Sphericity.

## Discussion

The present study aimed to determine the psychometric properties of the Persian version of PDM scale. Until the writing of the present paper, few studies have examined psychometric properties of the PDM scale in other languages [15, 18].

Exploratory factor analysis method was used to assess the construct validity of the Persian version of the questionnaire. In doing so, with respect to the eigenvalues higher than one and scree plot, two factors were extracted. This two-factor questionnaire with 10 questions can be easily and quickly practiced. Typically, in factor analysis, the percentage of communalities less than 0.5 is not acceptable, and the scores of such items should be eliminated; however, considering the importance of suicidal thoughts (item number seven), the research team decided not to eliminate item seven. The decision for maintaining such an important item is confirmed by statistical considerations in data analysis [19]. These results were similar to the results of factor analysis of the original questionnaire [15]. In a study by Hirsch et al. (2017), six questions were related to general depression and four questions were about obsessive-compulsive symptoms, while the question of suicide was not present among the factors [18]. The results of both studies were consistent with the present study. These results suggest that, despite the cultural differences in psychological variables in different communities, concerns about issues and problems related to pregnancy are universal.

Cronbakh’s Alpha for the PDM in this study was 0.75 and its ICC was 0.75(CI = 0.7 to 0.82).

These numbers confirm that PDM Scale is a reliable and coherent and applicable tool in the clinical environment. In the original study, Cronbach’s alpha for the English version of the PDM Scale, general distress and obsessive compulsive symptoms sub-scales was 0.88, 0.91 and 0.83, respectively [15], indicating a desirable reliability. Also, in another study by Hirsch et al. (2017), Cronbach’s alpha for the whole questionnaire, the general distress and the obsessive-compulsive subscales was 0.85, 0.84 and 0.80, respectively [18]. Although Cronbach Alpha correlation coefficient depends on the number of questions, the high Cronbach alpha does not show that tool is one dimensional; therefore, the low internal stability of the tool may be related to the low number of its questions [20]. On the other hand, the reliability of a questionnaire is indicative of the degree of measurement stability and proportionality of the items with each other [21]. According to the findings of this study, the psychometric properties of the Persian version of the PDM scale is desirable [22].

Study of criterion validity of the questionnaire showed that the results from the Persian version of PDM are highly correlated with the results from Cambridge pregnancy Distress Measure, which is as a valid and reliable tool. It seems that the Persian version of Postpartum Distress Measure evaluates constructs similar to the Cambridge pregnancy Distress Measure and it can be used as an acceptable tool for screening mental problems during postpartum period [23].

In the present study, the value of KMO was 0.811. KMO measure is an index for the sample size adequacy. Values greater than 0.6 are acceptable and the values closer to one are better [24]. Bartlett’s Sphericity test was used to determine the fitting of the model, which had a significant level. In Bartlett Sphericity test, if the significance value is less than 0.5, the ability to validate the data is confirmed. The two criteria mentioned are important indicators for confirming the adequacy of sampling and the ability to factor analysis of the items.

### Limitations of the study

Since the psychometric properties of the PDM scale in languages other than Persian was not already investigated, the researchers compared the psychometric properties of the Persian version of the questionnaire with only those of its original English version.

Moreover, in some women, distress during pregnancy imay be due to causes other than pregnancy. The main designers of PDM Scale have stated that the tool measures only the specific pregnancy outcomes; however, since our patients have not been evaluated and screened for physical or psychological problems, it is recommended to evaluate other psychological problems of the women in future studies, in order to yield more reliable results.

## Recommendations

“A cut off score is determined when a scale is criterion-referenced. In criterion-referenced scales, scores below the cut off represent the absence of the intended attribute. PDM scale is a norm-referenced scale and lower scores represent lower distress, not its absence. Hence, there was no need to determine the cut off score for this screening tool; however, if it is intended to apply it as a sensitive diagnostic tool, it is recommended to employ appropriate methods to determine its optimal cut off score”, so that the Persian version can be easily used for diagnosing clinical cases.

## Conclusion

According to the study results, the Persian version of PDM Scale has a coherent structure and acceptable psychometric properties. Applying this valid and reliable questionnaire, psychiatrists, other care providers and researchers can separately or simultaneously screen anxiety, depression and obsessive-compulsive disorder in women. It means that the PDM can be a helpful tool in identifying a broader range of postpartum distress, including obsessive-compulsive symptoms that were formerly neglected in clinical screening measures.

## Data Availability

The data sets generated and/or analyzed during the current study will not publicly available before publication of its pertinent manuscripts, but are available from the corresponding author on reasonable request.

## Abbreviations

CVI: Content Validity Index
CVR: Content Validity Ratio
DASS-21: Depression and Anxiety Stress Scale-21
EPDS: *Edinburgh* Postnatal *Depression Scale*
GAD-7: Generalized Anxiety Inventory
ICC: Intra-class Correlation Coefficient
IS: Impact score
KMO: Kaiser-Meyer-Olkin
PDM Scale: Postpartum Distress Measure Scale
SPD: Severe Psychological Distress
SPSS: Statiscal Package for social science
